# Three COVID-19 cases with a long-term viral shedding period in Tunisia

**DOI:** 10.11604/pamj.supp.2020.35.2.24950

**Published:** 2020-07-16

**Authors:** Cyrine Bennasrallah, Rania Bannour, Olfa Jlassi, Mariem Kacem, Manel Ben Fredj, Hela Abroug, Imen Zemni, Behaeddine Garrach, Rihab Bahri, Nesrine Charfeddine, Sayda Triki, Asma Belguith Sriha

**Affiliations:** 1 Department of Epidemiology and Preventive medicine, Fattouma Bouguiba University Hospital, University of Monastir, Monastir, Tunisia,; 2 Department of Occupational Health and Ergonomics, University of Monastir, Monastir, Tunisia,; 3 Department of Family Medecine, University of Monastir, Monastir, Tunisia

**Keywords:** COVID-19, SARS-CoV-2, outbreak, pandemic, shedding

## Abstract

Novel coronavirus disease (COVID-19) caused by severe acute respiratory syndrome-coronavirus-2 (SARS-CoV-2) has become a public health emergency of international concern. This was first emerged in Wuhan, Hubei Province, China, and then has become widespread all over the world. We report 3 cases (2 imported cases and 1 local case) with documented viral shedding (based on reverse transcription-polymerase chain reaction (RT-PCR) testing) of SARS-CoV-2 for 55, 59 and 63 days. Viral shedding duration was defined as the date of return from the COVID-19 pandemic countries for imported cases and from the first positive RT-PCR test for local cases, up to the second negative nasopharyngeal RT-PCR swab. These cases demonstrate that viral shedding after COVID-19 diagnosis can be prolonged.

## Introduction

In December 2019, an outbreak of severe acute respiratory syndrome coronavirus-2 (SARS-CoV-2) infection was detected in Wuhan, China. The large spread of SARS-CoV-2 has led to a massive pandemic, associated with an important morbidity and mortality [1]. Novel coronavirus disease (COVID-19) has spread to 216 countries, with more than 350,000 confirmed deaths worldwide [2]. According to some retrospective analyses, the median duration of SARS-CoV-2 RNA detection was 17 days (Interquartile Range [IQR], 13-22 days) as measured from illness onset [3]. We present 3 cases of COVID-19 with viral shedding for 55, 59 and 63 days.

## Patient and Observation

**Figure 1:**
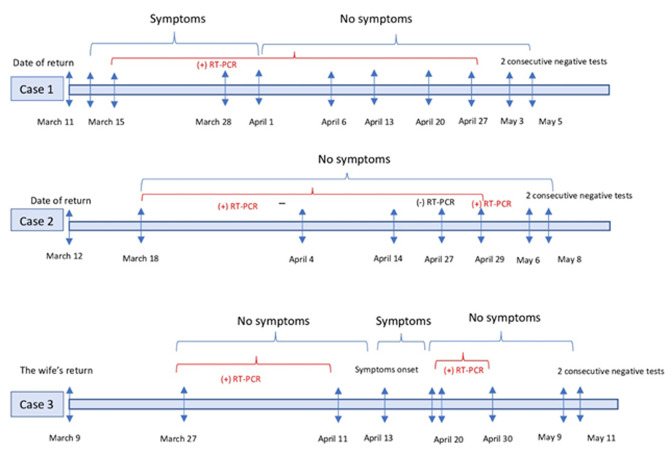
the dynamic of symptoms and severe acute respiratory syndrome-coronavirus-2 reverse transcriptase-PCR test results over time

**Case 1:** a 33-year-old Tunisian woman, returned from France on March 11, 2020. She was healthy and nonsmoker. The onset of symptoms occurred 3 days after return, on March 14th. The patient reported a dry cough and a shortness of breath. She called the emergency medical assistance from home. A subsequent nasopharyngeal swab RT-PCR test was positive for COVID-19 on March 15, 2020. This confirmation was performed in our National Reference Laboratory. The patient had no severe symptoms of infection; therefore, she was placed in home isolation. Case 1 was asymptomatic from April 1, 2020 but the RT-PCR test was still positive for SARS-CoV-2 virus on April 6,13,20 and 27. It turned negative on May 3 and 48 hours later on May 5. Then, the case was discharged from home quarantine 51 days after first RT-PCR, and after 2 consecutive nasopharyngeal swabs tested negative for SARS-CoV-2 (viral shedding duration 55 days) (Figure 1). Eleven nasopharyngeal swab RT-PCR tests were realized on March 18 within the contact tracing, revealing a COVID-19 transmission to a close family member (her mother). All his other contacts were asymptomatic for the following 2 weeks and were tested SARS-CoV-2 negative. The mother has a history of asthma and has been taking Seretide spray. During the isolation period, she presented fever, cough and a shortness of breath. The mother RT-PCR test was negative 9 days before her daughter.

**Case 2:** a 61-year-old male who returned from France on March 12, 2020 with a history of type 2 diabetes. He did not present any symptoms. Nasal swab was carried out due to the non-compliance of the patient in the quarantine. And, the presence of SARS-CoV-2 in respiratory specimens was detected by RT-PCR on 18 March 2020. The patient had no severe symptoms of infection and was placed in home isolation. Case 2 did not receive any treatment during his viral shedding. Repeat SARS-CoV-2 tests of nasopharyngeal swabs were negative on April 27 and positive on April 29. Then, the results of two continuous COVID-19 virus tests became negative on May 6 and 8. Thus, the patient was declared recovered (51 days after the first RT-PCR and 59 days of viral shedding duration) (Figure 1). The contact tracing revealed a second family case of COVID-19 that returned from France too on 8 March 2020 (the daughter). His daughter was declared cured on 29 April 2020 (40 days after the first RT-PCR and 52 days of viral shedding duration).

**Case 3:** it was a 75-year-old male who was living abroad and came back to Monastir, Tunisia in January 2020. He has a history of prostate cancer and hypertension. Three months later on March 9, 2020, his wife returned from France. His wife developed a dyspnea (80% oxygen saturation) and abdominal pain. Therefore, she was admitted into the infectious disease department. Regarding the symptoms and the epidemiological context, she was tested for SARS-CoV-2 and declared positive. The contact investigation around the patient revealed on March 27, 2020 a positive SARS-CoV-2 husband (case 3). The patient had no severe symptoms of infection and was placed in home isolation. Case 3 was asymptomatic at the beginning. The RT-PCR SARS-CoV-2 control on April 11th was positive. Two days after, the patient reported arthralgia, abdominal pain, vomiting and developed skin lesions. He became asymptomatic again on April 19. On April 20 and 30, the control RT-PCR SARS-CoV-2 was still positive. On May 9, 2020 the Rt-PCR was negative. The virus clearance was confirmed two days after, on May 11, 2020, 45 days after the first test (63 days of viral shedding duration) (Figure 1). His wife was recovered and discharged since April 4th.

## Discussion

We reported 3 cases of mild COVID-19 cases, for which the longest duration of viral shedding was 63 days from the first RT-PCR test. Viral shedding duration was defined as the date of return from the COVID 19 pandemic countries for imported cases and from the first positive RT-PCR test for local cases, up to the second negative nasopharyngeal RT-PCR swab. According to previous reports, median duration of viral shedding was 20.0 days (IQR 17.0-24.0) in survivors, but SARS-CoV-2 was detectable until death in non-survivors [4]. The longest reported viral shedding duration was 60 days among a Chinese woman [5]. Our cases recovered from a fairly mild episode of COVID-19, with no reported symptoms after 17 days for case 1, 51 days for case 2 and 22 days for case 3 from the first positive test but remain positive for SARS-CoV-2. It was showed that there is no correlation between the median duration of viral shedding and the severity of the disease similar to another study [6]. Our findings suggest that a proportion of clinical recovered patients still may be virus carriers. In fact, many studies have been reported on infections in close contacts of COVID-19 patients even after apparent clinical recovery of the source patients [7]. It remains unclear whether the persistent shedding is associated with prolonged infectivity [5].

According to a recent report, RNA can be detected long after the disappearance of infectious virus. The immune system can neutralize viruses but do not eliminate RNA, degrading slowly [8]. Further investigations based on larger cohorts are needed to characterize the duration of viral shedding and infectivity. Long viral shedding has important guiding significance for the isolation prevention measures. Consequently, asymptomatic and recovered patients should be in-home quarantine for an extended period of time of more than 14 days. In case 1, viral shedding was longer among the daughter than her asthmatic mother under corticosteroid. SARS-CoV-2 tests of nasopharyngeal swabs became negative 11 days after the daughter for case 2 and 31 days after the wife for case 3. Age, male gender and corticosteroid were factors associated with a long positive RT-PCR duration [3]. These findings were in line with what was reported in case 2 and 3 and in contrast with case 1. More studies with larger sample sizes are required to substantiate these findings.

## Conclusion

Further research is needed to characterize the prolonged viral shedding and infectivity. This will improve our knowledge about SARS-CoV-2 transmission and allow a better certainty around guidelines for contact tracing and quarantine periods.
